# Leiomyoblastome gastrique: à propos de trois cas

**DOI:** 10.11604/pamj.2015.20.42.4049

**Published:** 2015-01-15

**Authors:** Mountassir Moujahid, Issam Ennafaa, Ahmed EL Rhari, Issam Serghini, Khalid Chekoura, Moulay Hassan Tahiri

**Affiliations:** 1 Service de Chirurgie Générale, 5ème Hôpital Militaire, Guelmim, Maroc; 2 Service de radiologie, 5ème Hôpital Militaire, Guelmim, Maroc; 3 Service d'anesthésie et réanimation, 5ème Hôpital Militaire, Guelmim, Maroc

**Keywords:** Pathologie appareil digestif, tumeur, muscle lisse, estomac, léiomyoblastome, digestive tract pathology, tumor, smooth muscle, stomach, leiomyoblastoma

## Abstract

Le leiomyoblastome gastrique est une tumeur musculaire rare qui touche essentiellement l'adulte. Son développement est souvent exogastrique. Le diagnostic positif repose sur l'histologie et le traitement est basé sur la chirurgie. Nous rapportons trois cas de leiomyoblastome gastrique colligés dans le service de chirurgie générale au 5ème Hôpital Militaire. L’âge moyen des patients est de 47 ans; le motif de consultation était représenté par une hémorragie digestive et l'imagerie médicale a posé le diagnostic de masse tumorale dans tous les cas. Le traitement chirurgical consistait en une gastrectomie partielle et le compte rendu anatomopathologique a confirmé le leiomyoblastome gastrique dans les trois cas. Le siège de la tumeur a été posé par la fibroscopie oeso gastroduodénale, le traitement était chirurgical et les suites post opératoires étaient simples avec un contrôle par des fibroscopies répétitives sans aucun signe de récidive. Le leiomyoblastome gastrique est une tumeur rare. L’écho endoscopie joue un rôle primordial dans le diagnostic positif ainsi que dans l’évaluation de l'extension pariétale de ces tumeurs. Le traitement est essentiellement chirurgical.

## Introduction

Les tumeurs des fibres musculaires lisses du tube digestif sont rares qui touchent essentiellement l'adulte.et la localisation gastrique reste la plus fréquente. En effet, les tumeurs musculaires constituent moins de 2% des tumeurs du tractus digestif. Son développement est souvent exogastrique. Le diagnostic positif repose sur l'histologie et le traitement est basé sur la chirurgie. Nous rapportons trois cas de leiomyoblastome gastrique colligés dans le service de chirurgie du 5^ème^ Hôpital Militaire de Guelmim.

## Patient et observation

### Observation n°1

**Figure 1:**
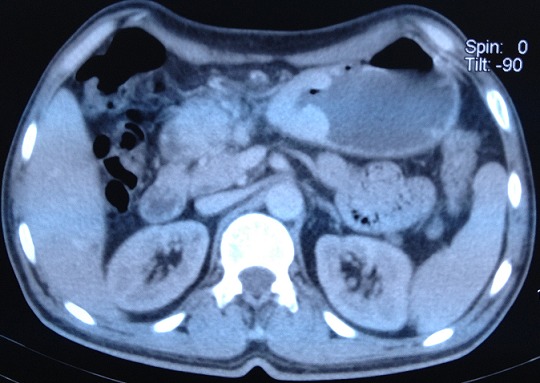
Épaississement de la région antrale étendu sur 81mm

**Figure 2:**
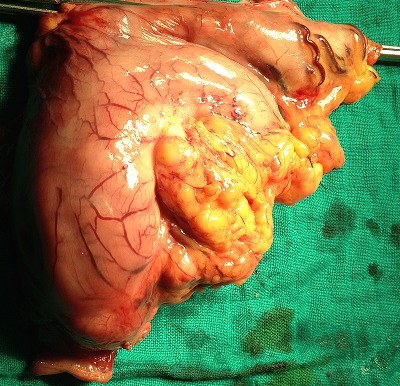
Pièce de gastrectomie partielle

Patient de 59 ans sans antécédents médicaux chirurgicaux admis le 6 /9/1996 pour une anémie sévère suite à des mélénas évoluant depuis six mois, l’état hémodynamique était stable avec une dyspnée, l'examen clinique mettait en évidence une sensibilité de la région épigastrique sans masse palpable ni d'hépato splénomégalie ni de circulation collatérale. Le toucher rectal était normal. La fibroscopie oeso gastro duodénale a montré un processus tumoral sous muqueux de 10 cm de diamètre sur la face antérieure de la petite courbure avec des ulcérations centrales contenant des caillots de sang. Les biopsies faites étaient revenues négatives. L’échographie abdominale a montré une masse au contact du foie gauche, le scanner a montré un épaississement de la région antrale étendu sur 81 mm environ sans adénopathies suspectes (Figure 1). Le malade à été opéré le 26/9/1996 par une voie médiane avec réalisation d'une gastrectomie partielle (Figure 2). L’étude anatomopathologique de la pièce avait conclu à la bénignité de la tumeur en faveur d'un leiomyoblastome gastrique. Les suites post opératoires étaient simples et le malade avait quitté le service au dixième jour. La surveillance endoscopique sur un recul de quinze ans n'a pas objectivé de récidives.

### Observation n°2

**Figure 3:**
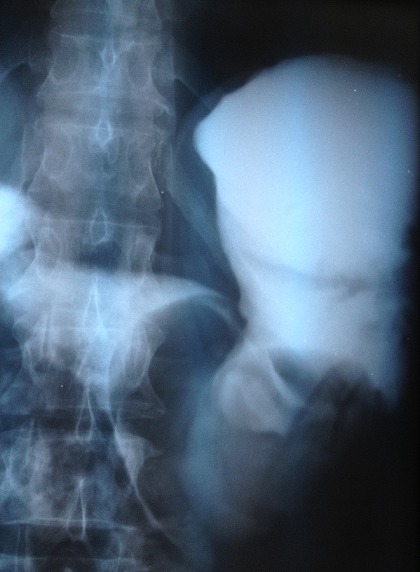
Transit à la gastrograffine montrant une tumeur médio gastrique de cm de diamètre

**Figure 4:**
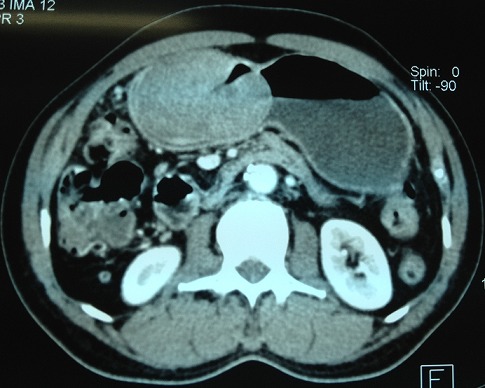
Épaississement pariétal de la région antrale avec développement d'une masse tissulaire intra luminale de 79x56mm de grand axe

**Figure 5:**
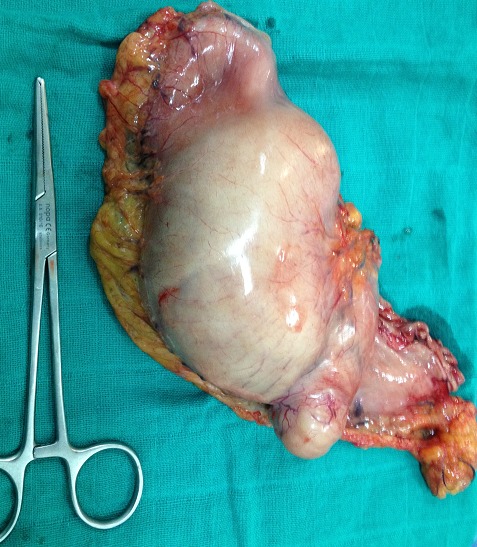
Pièce de gastrectomie partielle

Patient de 41 ans aux antécédents d’épigastralgie avec méléna évoluant depuis une année, admis en urgence pour un état de choc hypovolémique. La fibroscopie réalisée en urgence avait montré l'existence d'une tumeur gastrique de siège antral dont les biopsies n’étaient pas concluantes. La numération formule sanguine a montré une anémie hypochrome microcytaire à 4g /100ml. L’échographie abdominale a objectivé une masse gastrique arrondie pouvant correspondre à un léiomyome ou un fibroadénome. Le transit oeso gastro duodénal a montré que la tumeur était médiogastrique de 8 cm de diamètre d'aspect bénin sous muqueuse correspondant à un shwanome ou un fibrome (Figure 3). Le scanner abdominal a montré épaississement pariétal de la région antrale avec développement d'une masse tissulaire en intra luminale de densité tissulaire rehaussée après injection de produit de contraste, cette masse est bien limitée mesurant 79x56 mm de grand axe (Figure 4). Une gastrectomie partielle a été réalisée (Figure 5). Le compte rendu anatomopathologique a conclut en une prolifération cellulaire agencée en faisceaux longs, les cellules avaient un cytoplasme éosinophile et un noyau allongé sans atypies les mitoses étaient rares. Il n'a pas été vu de nécrose, les marges d'exérèses étaient saines, ce qui était en faveur d'un leiomyoblastome gastrique. Les suites étaient simples et la malade a quitté le service au dixième jour. Le recul est de 10 ans sans aucun signe de récidive.

### Observation n°3

Femme de 42 ans sans antécédents accusait depuis six mois une altération de l’état général avec dyspnée et une anémie hypochrome à 8g/100 ml ainsi que des épigastralgies. Le bilan des investigations a montré à la fibroscopie une grosse tumeur exulcérée de la petite courbure gastrique. Le bilan d'extension était normal. La patiente a subit une gastrectomie subtotale avec au compte rendu anatomopathologique une tumeur de type leiomyoblastome, sans localisation secondaire, de grade histologique 1. Les suites post opératoires étaient simples avec un contrôle annuel par des fibroscopies répétitives sans aucun signe de récidive sur un recul de trois ans.

## Discussion

La première description de cette tumeur musculaire lisse remonte à 1960 par Martin [1]. Stout a proposé le terme de Leiomyoblastome en 1962. C'est une tumeur d'origine mésenchymateuse qui représente 8à15% des tumeurs musculaires de l'estomac [2]. L’âge de prédilection de ces lésions est au delà de la cinquantaine avec des exceptions chez l'enfant de 7 ans [3]. C'est l'homme qui est touché deux fois plus que la femme [2]. Le leiomyoblastome se localise dans 80% des cas au niveau de l'estomac et l'atteinte antrale est la plus fréquente 75% des cas. C'est une tumeur qui peut être exogastrique aussi bien qu'endogastrique. Dans ce dernier cas elle est sous muqueuse, ce qui explique les difficultés de diagnostic endoscopique. Leur présentation clinique est variable et dépend de la taille et du siège de la tumeur; en effet les tumeurs intramurales sont les plus souvent asymptomatiques alors que la clinique des tumeurs exogastriques se résume à des signes d'emprunte des organes de voisinage. Les hémorragies digestives ou les hémopéritoines sont les circonstances de découverte les plus fréquentes, plus de 50% des cas [4]. Ce n'est que rarement qu'on arrive à palper une masse de siège épigastrique. Chez la jeune fille le leiomyoblastome peut faire partie de la triade de Carney, qui n'est complète que dans 27% des cas [5, 6]. (Le leiomyoblastome gastrique, chondrome pulmonaire et paragangliome sécrétant le plus souvent extrasurrénalien). La fibroscopie gastrique objective la tumeur si elle est endogastrique et permet de faire des biopsies qui reviennent souvent négatives puisque les lésions sont sous muqueuses. Le transit oeso gastro duodénal révèle la lésion endogastrique sous forme de lacune avec parfois une ulcération à son centre réalisant l'aspect d'un ménisque. Le diagnostic lésionnel a largement bénéficié de l'imagerie médicale essentiellement la tomodensitométrie. L’échographie abdominale peut retrouver une masse hétérogène sans déterminer son siège exact. Le scanner abdominal en plus de son rôle dans le bilan d'extension, permet un diagnostic topographique précis ainsi que la nature tissulaire de la tumeur par l’étude des densités. Le diagnostic densitométrique de masse extrinsèque repose sur trois éléments de la sémiologie radiologique [7, 8]: agrandissement de la cavité gastrique; déformation fixe constante; image d'adhérence de la paroi à la masse. A noter que la nature myogène ou neurogène de la tumeur peut être étudiée à la tomodensitométrie après injection de produit de contraste [2, 4–8]. L’écho endoscopie est plus fiable et permet de différencier entre compression extrinsèque et tumeur intra pariétale et permet aussi des biopsies écho guidées affirmant le diagnostic histologique dans 75% des cas [9]. Le diagnostic différentiel se pose avec les lymphomes, les kystes et les abcès de la région épigastrique. Mais c'est le diagnostic de leiomyosarcome qui doit être éliminé, chose qui ne peut être faite que par l’étude histologique minitueuse de la pièce d'exérèse opératoire complète. Du point de vue histologique la cellule constituant le leiomyoblastome présente trois caractéristiques à retenir: absence totale de myofibrille; rareté des mitoses; importante anisocaryose.

Le potentiel malin de ces tumeurs est certain mais difficile à évaluer et repose essentiellement sur la taille de la tumeur (la transformation maligne n'est possible que pour les tumeurs atteints plus de deux centimètres) [10, 11]; le nombre de mitoses (plus de dix mitoses par 50 cellules), la vascularisation excessive et l'existence des zones de nécrose [12]. Mais la malignité ne peut être confirmé que par l'existence de métastases concomitantes [13, 14]. Sur le plan thérapeutique, l'exérèse chirurgicale de ces tumeurs est justifiée devant la lenteur d’évolution, l'apparition de métastases qui surviennent dans 10 à 20% des cas parfois tardivement [5, 8, 10, 15, 16]. En pratique la prise en charge de ces malades dépens surtout de quatre facteurs à savoir les circonstances de découverte, la taille de la tumeur, ses caractéristiques endoscopiques et l’âge du malade. En effet lorsque la traduction clinique est sous forme d'hémorragie digestive susceptible de récidiver, l'exérése de la tumeur est impérative. Une taille tumorale supérieure à deux centimètre ou des caractéristiques endoscopiques en faveur de la malignité, tels que l'existence de zones nécrosées et irrégulières avec une extension locorégionale; impose un geste chirurgical ou une surveillance rapprochée pour ne pas laisser évoluer un leiomyosarcome méconnu [17–19]. Enfin l’âge du malade est un facteur décisionnel relatif. Les patients de moins de 60 ans doivent être opérés alors qu'au delà on a le choix entre une chirurgie d'exérèse ou une surveillance endoscopique adéquate. Le geste chirurgical dépend du siège, de la taille et des résultas de l’étude extemporanée de la tumeur. Donc on peut être amené à pratiquer une tumorectomie en laissant une marge de sécurité de tissus sains de 2 cm, ou une gastrectomie partielle ou totale. L’évolution reste l'un des éléments principaux pour démontrer à posteriori la bénignité ou la malignité de ces tumeurs. La dégénérescence du Leiomyoblastome peut se voir dans une proportion allant de 0 à 15% des cas surtout pour les tumeurs à développement exogastrique avec un diamètre supérieur à 2cm dont le risque peut atteindre 55% des cas [18–20].

## Conclusion

Le leiomyoblastome gastrique est une tumeur rare. L'hémorragie digestive est le principale symptome. L’échoendoscopie joue un rôle primordial dans le diagnostic positif ainsi que dans l’évaluation de l'extension pariétale de ces tumeurs. Le traitement est essentiellement chirurgical. Le pronostic reste encore à établir, car une tumeur rassurante au départ doit inciter à une surveillance adéquate à long terme car la malignité peut compliquer l’évolution de cette tumeur de façon imprévisible.
